# Patterns of complementary and alternative medicine use in pediatric patients with inflammatory bowel disease

**DOI:** 10.1002/jpn3.70252

**Published:** 2025-11-06

**Authors:** Katharina Guilcher, Valerie Pittet, André Moser, Laetitia‐Marie Petit, Klaas Heyland, Christian Braegger, Andreas Nydegger, Luca Garzoni, Susanne Schibli, Johannes Spalinger, Franziska Righini‐Grunder, Christiane Sokollik, the Swiss IBD Cohort Study Investigators

**Affiliations:** ^1^ Department of Pediatrics, Inselspital, Division of Pediatric Gastroenterology, Hepatology and Nutrition Bern University Hospital University of Bern Bern Switzerland; ^2^ Department of Epidemiology and Health Systems Center for Primary Care and Public Health (Unisanté) – University of Lausanne Lausanne Switzerland; ^3^ CTU Bern University of Bern Bern Switzerland; ^4^ Division of Pediatric Gastroenterology University Hospital of Geneva Geneva Switzerland; ^5^ Department of Pediatrics Cantonal Hospital Winterthur Winterthur Switzerland; ^6^ Division of Pediatric Gastroenterology University Children′s Hospital of Zurich Zurich Switzerland; ^7^ Division of Pediatric Gastroenterology Lausanne University Hospital and University of Lausanne Lausanne Switzerland; ^8^ Pediatric Gastroenterology, Clinique des Grangettes Geneva Switzerland; ^9^ Division of Pediatric Gastroenterology, Children′s Hospital of Lucerne Lucerne Switzerland

**Keywords:** biologicals, Crohn′s disease, diet, treatment, ulcerative colitis

## Abstract

**Objectives:**

Inflammatory bowel disease (IBD) constitutes a challenging and long‐lasting burden for pediatric patients. The management of IBD is multifaceted involving conventional medication but also dietary measures. An increasing number of patients are turning to complementary and alternative medicine (CAM) in their quest to alleviate IBD‐related symptoms and improve their overall well‐being. We therefore investigated the patterns of CAM use and associated patients' and disease characteristics to identify indicators of CAM use in pediatric IBD patients in Switzerland.

**Methods:**

Pediatric and adolescent patients enrolled in the Swiss IBD Cohort Study, a nationwide prospective cohort study, completed a questionnaire about CAM and supplementation use as well as dietary restrictions. Associations of CAM use with disease characteristics including conventional medication and patient‐recorded outcomes were analyzed.

**Results:**

A total of 111 pediatric IBD patients (59 Crohn′s disease, 41 ulcerative colitis, 11 IBD unclassified) answered the questionnaire. Sixty‐five percent (72/111) of patients used at least one CAM since diagnosis, 73% (81/110) took vitamins and micronutrient supplements and 53% (59/111) followed dietary restrictions. The latter was in 68% self‐imposed and significantly associated with CAM use in multivariable models (odds ratio [OR]: 4.48; 95% confidence interval [CI]: 1.84–10.92). CAM use was not associated with IBD subtype, need for biologicals or extraintestinal manifestations. Health‐related quality of life (HRQOL) and disease activity did not differ between CAM and no‐CAM users.

**Conclusion:**

CAM use as well as self‐imposed dietary restrictions are frequent in IBD patients, whereby both are closely interrelated. While CAM users do not have higher HRQOL compared to no‐CAM users, they subjectively report an improvement in health associated with CAM use.

## Abstract (highlights)

**What is Known:**

- The demand of complementary and alternative medicine (CAM) in acute and chronic diseases is increasing.
- Data about the use in pediatric inflammatory bowel disease (IBD) are scarce.

**What is New:**

- The prevalence of complementary and alternative medicine use is high in pediatric IBD.
- CAM is often associated with dietary restrictions, 68% of which are self‐imposed.
- Treating physicians should be aware of this close correlation and integrate these facts into their treatment strategy.

## Graphical abstract

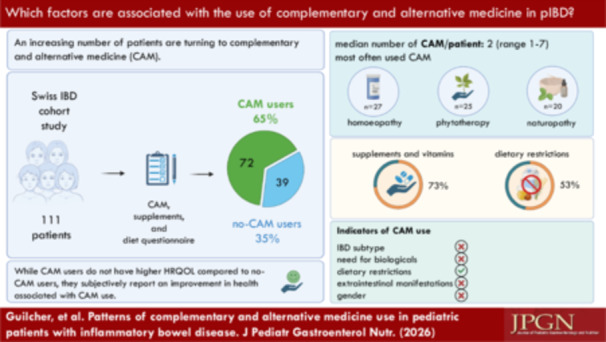

## 1 INTRODUCTION

Inflammatory bowel disease (IBD) is a chronic relapsing inflammatory disorder of the gastrointestinal tract with the two main subtypes Crohn′s disease (CD) and ulcerative colitis (UC). Currently, they lack a definitive cure in most cases. Over the disease course, treatment escalations are often necessary despite the availability of a broad armamentarium of conventional medications.^1^, ^2^ Disease burden of IBD therefore is high and related with poorer quality of life compared to healthy individuals.^3^ Given these challenges, patients often explore alternative treatment options. Notably, the use of complementary and alternative medicine (CAM) among adults with IBD is high.^4^

CAM is defined as a group of therapeutic and diagnostic approaches that are used in combination with or as an alternative to conventional medicine. Classical CAM includes a wide range of practices such as acupuncture, massage therapy and herbal remedies. In the view of the general population, the difference between classical CAM, probiotics, as well as vitamin and micronutrient supplementation is overlapping. Governments have established national centers for complementary and integrative health to support the research and provide information of those approaches.^5^

However, real‐life data about CAM use, dietary supplementations, and dietary restrictions in relation to the disease severity in IBD and especially pediatric IBD are scarce. Some, albeit mostly qualitatively weak, evidence of the benefit of CAM in IBD exists.^6^, ^7^ The best evidence is available for probiotics. For example, a multistrain probiotic containing eight different probiotics is recommended for maintaining remission for pouchitis in pediatric and adult patients with UC.^1^ Additionally, in patients with mild to moderate UC an integrative approach with probiotics in combination with conventional therapy showed some beneficial effect.^8^, ^9^ One well‐established dietary approach in pediatric CD is the use of exclusive enteral nutrition (EEN) for induction of remission.^2^ As adherence is the main obstacle for EEN, increased attention is paid to whole‐food‐based diets, such as CD exclusion diet (CDED) and CD treatment with eating diet (CD‐TREAT).^10^, ^11^ Whilst replicating the composition of EEN by excluding certain dietary components, these diets showed not only improved patients adherence but also effectiveness in inducing remission.^12^ In contrast to such specific diets tailored for treating IBD, there is no evidence for a beneficial effect of other special diets (e.g., gluten‐free, low carbohydrate) or dietary supplements (e.g., Omega 3, peppermint oil) on IBD disease course.^13^ However, self‐imposed dietary restrictions are commonly reported in adult IBD patients.^14^, ^15^, ^16^ Reasons for diet restrictions were “benefits for my health and well‐being,” “benefits for my IBD” or perception of food intolerances. In a questionnaire based pediatric study, more than one‐third of patients and parents believed that diet plays a role in the course of IBD. About 60% of patients and 40% of parents reported some dietary changes after the IBD diagnosis.^17^ One concern with self‐imposed diets are the possible occurrence of nutritional deficiencies and imbalances.^18^

The aim of our study was to define real‐life CAM use in pediatric IBD in Switzerland and to identify indicators for CAM use. Additionally, we evaluated supplementation and dietary restrictions and associated classical CAM use with patients' and disease characteristics, medical therapies and health‐related quality of life (HRQOL). We also evaluated perceptions about CAM use from both, patients and parents.

## 2 METHODS

### 2.1 Ethics statement

The study was approved by the ethics committee of the cantons or regions of Switzerland in which patients were included (SIBDCS Project N°2019‐09). Written informed consent of patients or caregivers to participate in the SIBDCS including the CAM‐SD, complementary and alternative medicine, supplements, and diets (CAM‐SD) questionnaire was obtained.

### 2.2 Patients

Patients under the age of 18 enrolled in the national Swiss IBD Cohort Study (SIBDCS) were recruited prospectively to complete the CAM‐SD questionnaire between December 2019 and August 2021.

The aims and methodology of the SIBDCS have been described previously in detail.^19^ In short, the SIBDCS is a multicenter cohort study which captures at enrollment and at subsequent yearly follow‐ups disease‐related information such as IBD subtype, disease behavior (penetrating, stricturing), complications, diagnostic work‐up including clinical activity scores (weighted pediatric CD Activity Index [wPCDAI] for CD^20^ and Pediatric Ulcerative Colitis Activity Index [PUCAI] for UC/IBD‐unclassified [IBD‐U]),^21^ laboratory values, imaging and endoscopies as well as extra‐intestinal manifestations, medications, medication side effects, need for surgery and hospitalizations.

As part of the SIBDCS, patients between ages 9–17 additionally answer the validated IMPACT III (CH) questionnaire,^22^ which evaluates the HRQOL of pediatric IBD patients. Total scores and subscores are calculated as described by Werner and colleagues.^22^ A higher score indicates better HRQOL. The score ranges are as follows: Total score 22–110, emotional functioning 8–40, body image 4–20, social functioning 3–15, IBD symptoms 7–35.

### 2.3 CAM‐SD questionnaire

We developed the CAM‐SD questionnaire based on previously published CAM questionnaires in pediatric patients^23^ and adapted it to the Swiss environment by using the Swiss National Survey on CAM.^24^ Patients and/or parents completed the questionnaire in personal interviews at their yearly SIBDCS follow‐up. Questions included whether patients use the following therapies currently or have used them in the past 12 months: Herbal medicine, homeopathy, alternative professional care encompassing body and mind practices (e.g., hypnosis, massage, and meditation), manual therapies (e.g., osteopathy and chiropractic), traditional Chinese medicine (TCM) and other therapies such as acupuncture, kinesiology, naturopathy, shiatsu, autogenic training, neural therapy, bioresonance therapy, and anthroposophic medicine. If one of these CAM were used, patients were allocated to “CAM user.” All other patients were allocated to “no‐CAM users.”

The CAM‐SD questionnaire also encompassed questions about the use of supplements including micronutrients and vitamins, probiotics as well as dietary restrictions. Dietary restrictions included avoidance or reduction of lactose, gluten, fiber, fat, spices, sugars, or seeds and nuts. In cases of a gluten‐free diet we requested whether this was due to diagnosis of celiac disease. Nutritional therapies such as EEN, CDED or CD‐TREAT were not part of this questionnaire.

For micronutrient and vitamin supplementation, we specifically asked whether they were prescribed by the patient′s treating physician.

Additionally, the questionnaire explored reasons for dietary restrictions, motivation for CAM use, cost awareness of CAM treatment, and whether the treating physician was informed about the CAM use.

Patients and parents were separately asked about their perceived subjective influence of CAM in regard to the patients' health (a four‐point scale was given “improvement,” “unchanged,” “worsening,” “don′t know”). If the patient was not capable to answer this question, only parents were asked.

An interval of ±3 months between CAM‐SD questionnaire and the follow‐up data in the SIBDCS was accepted for analysis.

Due to the SIBDCS set‐up, we were unable to determine whether all eligible patients were actually invited to complete the additional questionnaire, whether any declined to do so, and what reasons led to their refusal. To reduce a possible selection bias, we compared the baseline characteristics of patients with and without a CAM‐SD questionnaire.

### 2.4 Statistics

We describe the study population by frequencies (*n*), percentages (%), median, and interquartile range. We compared groups using chi‐squared test or Fisher's exact test for categorical variables and Wilcoxon rank sum test or Kruskal±Wallis test for continuous variables. We modeled the indicator CAM versus no‐CAM user using univariable (only one variable used as indicator) and multivariable (all variables jointly used as indicators) logistic regression models. In multivariable models, we adjusted for variables: biologicals ever used (1 vs. 0, 2 and more vs. 0), diagnosis (CD vs. UC/IBD‐U), dietary restrictions (yes vs. no), any extraintestinal manifestations and gender (female vs. male). Those variables were chosen a priori after consultation with clinicians. We reported odds ratios (ORs) with 95% confidence intervals (CIs). All *p*‐values were two‐sided, and *p*‐values < 0.05 were considered statistically significant. All analyses were performed using R version 4.2.3 (R Core Team).

## 3 RESULTS

### 3.1 Characteristics of patients

We received 111 CAM‐SD questionnaires of 183 eligible pediatric and adolescent IBD patients of the SIBDCS, which corresponds to a rate of 60.6%. There were no significant differences between the baseline characteristics of patients with and without a CAM‐SD questionnaire (Supporting Information S1: Table S1).

Of the 111 with a CAM‐SD, 72 (65%) were CAM users and 39 (35%) no‐CAM users. Baseline characteristics are summarized in Table 1. The two groups had similar baseline characteristics across all captured variables, including demographic characteristics, disease phenotype, extraintestinal manifestations, linguistic region (French and German‐speaking part of Switzerland), family history of IBD, and medication side effects.

**Table 1:** Baseline characteristics of the study population and conventional IBD medication before and at CAM‐SD questionnaire.

Characteristic	Total	CAM	No‐CAM	*p* value
Patient number	111 (100)	72 (65)	39 (35)	
Age at diagnosis, years, median (IQR)	10.0 (7.0, 13.0)	10.0 (6.5, 13.0)	11.0 (7.0, 13.0)	0.372
Age at CAM‐SD, years, median (IQR)	14.0 (12.0, 16.0)	14.0 (12.0, 16.0)	14.0 (12.0, 16.0)	0.841
Disease duration at CAM‐SD, years, median (IQR)	4.0 (3.0, 6.0)	4.0 (3.0, 6.0)	3.0 (2.0, 5.0)	0.259
Sex, female	54 (49)	33 (46)	21 (54)	0.420
Diagnosis, CD/UC/IBD‐U	59 (53)/41 (37)/11 (10)	37 (51)/25 (35)/10 (14)	22 (56)/16 (41)/1 (3)	0.168
Extraintestinal manifestations	55 (50)	37 (51)	18 (46)	0.598
Region				0.7
French‐speaking	43 (39)	29 (40)	14 (36)	
German‐speaking	68 (61)	43 (60)	25 (64)	
Family history of inflammatory bowel disease	13 (12)	6 (8.3)	7 (18)	0.214
Dietary restrictions	59 (53)	48 (67)	11 (28)	<0.001
Medication side effects	11 (10)	9 (12)	2 (5)	0.323
Medications ever used during follow‐up until CAM‐SD				
Immunomodulator (azathioprine, 6‐mercaptopurine, methotrexate)	81 (73)	53 (74)	28 (72)	0.837
Systemic steroids	73 (66)	52 (72)	21 (54)	0.051
Oral 5‐ASA	61 (55)	41 (57)	20 (51)	0.567
Topical 5‐ASA	29 (26)	21 (29)	8 (21)	0.322
Budesonide	12 (11)	9 (12)	3 (8)	0.535
Biologicals (Infliximab, Adalimumab, Certolizumab, Vedolizumab, Golimumab, Ustekinumab)				0.053
Never biologicals	46 (41)	25 (35)	21 (54)	
1 biological	49 (44)	34 (47)	15 (38)	
2 biologicals	8 (7)	5 (6.9)	3 (7.7)	
3 or more biologicals	8 (7.2)	8 (11)	0 (0)	
Anti TNF alpha inhibitor (Infliximab, Adalimumab, Certolizumab)	64 (58)	46 (64)	18 (46)	0.071
Medications used at CAM‐SD				
Immunmodulator	40 (36)	26 (36)	14 (36)	0.982
Systemic steroids	22 (20)	14 (19)	8 (21)	0.893
Anti TNF alpha inhibitor (Infliximab, Adalimumab, Certolizumab)	42 (38)	27 (38)	15 (38)	0.921

*Note*: Conventional IBD medication before and at CAM‐SD are listed. Extraintestinal manifestations include arthritis, ophthalmological involvement, pyoderma gangrenosum, erythema nodosum, aphthous oral ulcers, ankylosing spondylitis, primary sclerosing cholangitis. Medications used in ≤8 patients (antibiotics, Sulfasalazine, Calcinorininhibitors [Tacrolimus, Ciclosporine], topical steroids and small‐molecules [Tofacitinib]) showed no statistically difference in use and are therefore not listed separately. If not specified total number and percentage was given *n* (%).

Abbreviations: 5‐ASA, 5‐aminosalicylic acid; CAM‐SD, complementary and alternative medicine, supplements, and diets; CD, Crohn′s disease; IBD, inflammatory bowel disease; IBD‐U, inflammatory bowel disease unclassified; IQR, interquartile range; TNF, tumor necrosis factor; UC, ulcerative colitis.

Additionally, there were no differences observed when analyzing CD and UC/IBD‐U separately including disease extent and behavior as well as disease activity at CAM‐SD (Supporting Information S1: Table S2 and S3).

### 3.2 CAM use

In the 72 CAM‐users, the median number of CAM used was 2 with a range from 1 to 7. Most reported CAM was homeopathy (27/101, 26.7%) and herbal medicine (27/108, 25%), followed by naturopathy (20/97, 20.6%), massages (20/101, 16.8%), and kinesiology (11/95, 10.2%). A detailed listing of different CAM use is provided in Supporting Information S1: Table S4.

The spectrum of phytotherapy was broad with curcumin being the most frequently used. 47% (7/15) patients who used Curcumin had the diagnosis of CD, 33% (5/15) UC and 20% (3/15) IBD‐U. Only one patient had a prescription by a physician for curcumin. Psychotherapy was common with 20% (22/108), in 12 patients it was prescribed by a physician and 6 initiated it by themselves.

### 3.3 Supplementation

Vitamins or dietary supplements were taken by 74% (81/110) of patients. Precise details are provided in Supporting Information S1: Table S5. 69% (60/86) of patients, who took Vitamin D supplements, had a prescription. Multivitamins and other supplements were mostly taken without prescription. Micronutrient supplementation was taken by 61% (68/111) of patients. In most cases, micronutrient supplementation was prescribed by a physician, especially iron. 33% (36/109) of patients reported using probiotic supplementations, of whom 69% had a prescription.

### 3.4 Dietary restrictions

Dietary restrictions were reported by 53.1% (59/111) of the patients, 29/59 with CD and 30/59 with UC/IBD‐U. Of those 61% (36/59) reduced milk products and/or lactose, 25.4% (15/59) gluten and 23.7% (14/59) spicy food. One patient on a gluten free diet had celiac disease.

The main reason to follow a restricted diet was to alleviate symptoms, which was reported by 44% (26/59) of the patients. In 26% (18/59) of cases, a physician recommended the dietary restrictions, and in 68% (40/59) of cases, they were self‐imposed.

### 3.5 Indicators of CAM use

In multivariable analysis, patients with dietary restrictions had a significantly higher chance of using CAM compared to those without dietary restrictions (Table 2). Patients, who had ever used biologicals, used CAM numerically more often than patients without biological therapy. This was even more pronounced when patients had received two or more biologicals.

**Table 2:** Logistic regression model for indicators of CAM use (univariable and multivariable analysis).

	Unadjusted OR (95% CI)	*p* value	Adjusted OR (95% CI)	*p* value
Biologicals ever used: 1 versus 0	1.90 (0.82–4.41)	0.133	1.66 (0.66–4.19)	0.284
Biologicals ever used: 2+ versus 0	3.64 (0.91–14.51)	0.067	1.87 (0.41–8.54)	0.422
Dietary restriction: yes versus no	5.09 (2.17–11.94)	<0.001	4.48 (1.84–10.92)	0.001
Extraintestinal manifestation	1.23 (0.57–2.69)	0.599	0.99 (0.42–2.38)	0.990
Female gender	0.73 (0.33–1.59)	0.421	0.73 (0.31–1.71)	0.470

*Note*: Biologics include Infliximab, Adalimumab, Certolizumab, Vedolizumab, Golimumab, and Ustekinumab. Extraintestinal manifestations include arthritis, ophthalmological involvement, pyoderma gangrenosum, erythema nodosum, aphthous oral ulcers, ankylosing spondylitis, and primary sclerosing cholangitis. “Ever used” refers to a medication of IBD since diagnosis until the CAM‐SD (complementary and alternative medicine, supplements and diets) questionnaire.

Abbreviations: CAM, complementary and alternative medicine; CAM‐SD, complementary and alternative medicine, supplements, and diets; CI, confidence interval; OR, odds ratio.

### 3.6 HRQOL and patient‐reported experience with CAM use

HRQOL information was available from 62 out of 102 eligible children and adolescents. There was no significant difference in disease‐specific overall IMPACT III (CH) score between CAM and no‐CAM users (median 98 vs. 99, respectively, *p* = 0.705) (Table 3). Also, when stratified by IMPACT III (CH) subgroups (emotional functioning, body image, social functioning, and IBD symptoms), the analysis revealed no significant difference between CAM and no‐CAM users.

**Table 3:** IMPACT III (CH) scores and subscores.

HRQOL	Total	CAM	No‐CAM	*p* value
Number of IMPACT III (CH) questionnaires, *n*	62	40	26	
IMPACT III (CH) total score	98 (91, 102)	99 (90, 104)	98 (92, 100)	0.705
Emotional functioning subscore	35.0 (31.0, 38.0)	35.0 (30.5, 37.0)	35.0 (31.0, 38.0)	0.725
Body image subscore	16.00 (14.00, 18.00)	16.00 (14.00, 18.00)	16.00 (14.00, 17.00)	0.376
Social functioning subscore	15.00 (15.00, 15.00)	15.00 (14.00, 15.00)	15.00 (15.00, 15.00)	0.511
IBD symptoms subscore	33.0 (31.0, 34.0)	32.5 (30.0, 35.0)	33.0 (31.0, 33.0)	0.584
Unknown, *n*	40	31	9	

*Note*: CAM users compared to no‐CAM users, if not otherwise specified, values are reported as median (IQR).

Abbreviations: CAM, complementary and alternative medicine; HRQOL, health‐related quality of life; IBD, inflammatory bowel disease; IQR, interquartile range.

**Figure 1:**
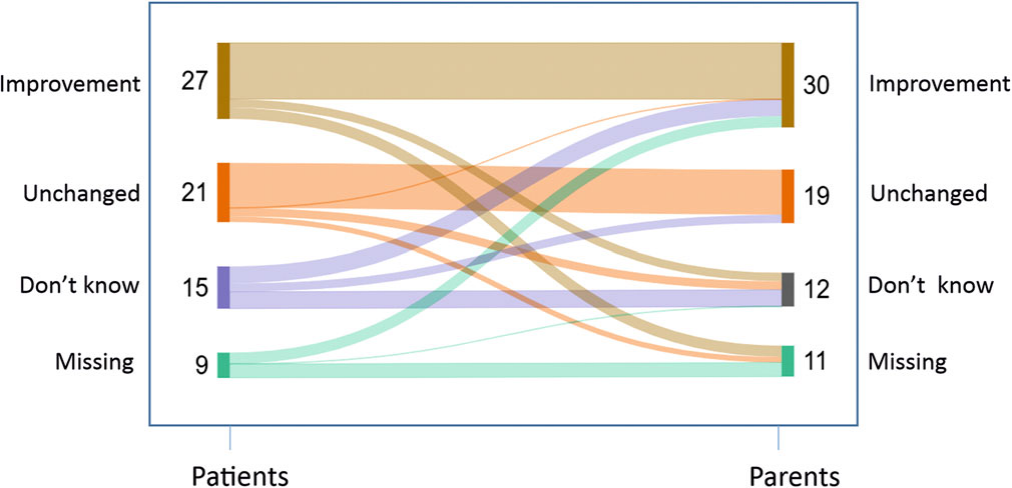
Perception of the influence of CAM on disease status or symptoms. CAM, complementary and alternative medicine.

In the four‐point scale about subjective influence of CAM use in the CAM‐SD questionnaire, 27/72 (38%) of patients indicated an improvement correlated to CAM use. 25/72 (34%) patient‐parent pairs agreed in their view that the use of CAM improved their health (Figure 1). “Worsening” of health in correlation to CAM use was not reported neither by patients nor parents. HRQOL for patients was independent of subjective impression (*p* = 0.161).

### 3.7 Motivation, information sources, cost coverage, and communication

Constituting 55.6% of responses, the primary driver for patients and families to use CAM was the anticipation of potential benefits without associated risks of side effects. Most patients sought information about CAM from their social circles, with 53% relying on friends and families. Notably for Switzerland, certain CAM therapies, including anthroposophical medicine, homeopathy, herbal medicine, and TCM, are eligible for coverage by mandatory basic health insurance. In our cohort, insurance covered 33% of the costs (Supporting Information S1: Table S1). Seventy‐six percent of families disclosed their CAM use, at least partially, to their physician.

## 4 DISCUSSION

In our study, 67% of pediatric and adolescent IBD patients reported using CAM, 73.6% of patients took vitamins or dietary supplements and 53% restricted their diet. These percentages are in accordance with other studies in pediatric IBD which report 41%–84% of CAM use.^23^, ^25^ In comparison, the prevalence of CAM use in the general population in Switzerland in 2017 was only 24.7%.^24^ There seems to be a general trend of increased CAM use of patients with gastrointestinal diseases over the last decades. One study in pediatric gastroenterology noted a doubling of CAM use from 36% in 2001 to 69% in 2008.^26^ Previous studies in adult patients with IBD showed geographical disparities with higher rates of CAM use in Northern America than in Europe.^27^ Noticeably, originating from the French or German‐speaking part of Switzerland made no difference in CAM use in our study.

Interestingly, most pediatric IBD patients who used CAM were exposed to more than 1 CAM in our and other pediatric cohorts.^28^ It can be assumed that there is a general openness to alternative therapy options in these families. Most families seem to educate themselves about CAM, but a better patient information and education is required. For example, curcumin, a component of turmeric, was used without a prescription in all but one patient in our cohort. There is some evidence for the benefit of curcumin for pediatric UC and it is therefore recommended as a possible add‐on therapy in mild‐to‐moderate disease.^1^, ^29^ However, two‐thirds of our patients who used curcumin had CD and not UC.

One previous study described that moderate/severe disease activity was associated with CAM use.^23^ In our study, we found higher ORs, however, statistically not significant, for CAM use in patients under biological therapy. Patients who need biological therapy generally suffer from a more severe phenotype, may have failed one or more conventional therapy approaches or may have encountered a drug related adverse effect.^30^ This may explain our and others' finding that parents are receptive to CAM use because they view CAM as a therapy with potential benefits and relatively small concern regarding adverse events.^28^ There seems to be a general underestimation of risks of CAM use and especially for herbal medicine.^31^, ^32^ Examples of negative interactions of CAM with conventional medication are St. John′s wort and *Ginkgo biloba*, which can reduce the efficacy of immunosuppressants (e.g., calcineurin inhibitors) and anticoagulants.^33^, ^34^ CAM also has a risk of contamination and impurity. One study found alarmingly high concentrations of organophosphorus insecticides in samples of medicinal herbs consumed by newborns and children.^35^ Furthermore, a systematic review found 79 types of herbs or herbal components which were related to herbal induced liver injury and even led to liver transplantation in 6.6% of these patients.^36^ Technical risks e.g. for acupuncture include severe complications such as pneumothorax and hepatitis.^37^, ^38^, ^39^

An independent indicator of CAM use in our study was dietary restrictions. Within our cohort, more than 50% of patients implemented dietary restrictions of which 68% were self‐imposed. Among the various dietary restrictions reported, the most frequent restriction was the reduction or avoidance of lactose in 61% of our patients. Lactose avoidance is also commonly observed in adults with IBD particularly during active disease, with reported rates of 40%.^40^ One explanation is a significantly reduced lactase activity found in duodenal biopsies and H2 breath testing in adult patients with active CD compared to patients in remission.^41^ Good data on lactase activity and lactose tolerance testing in pediatric and adolescent IBD patients are lacking, as most studies compared these patients to those with functional abdominal pain rather than to healthy controls.^42^ One additional point worth to raise is the risk of macro‐ and micronutrients as well as vitamin deficiency when following a restricted diet. Pediatric IBD patients are per se at risk for malnutrition and therefore, unnecessary elimination of nutrients from the diet should be discouraged as this may aggravate deficiencies.^43^ Consequently, physicians should always ask about self‐imposed dietary restrictions among CAM users and vice versa.

Around 40% of patients in our study reported improvement of their health associated with CAM use. Interestingly, our analysis, with the limitation of only one time point recorded, did not reveal a statistically significant difference in HRQOL between CAM and no‐CAM users. One reason may be that the HRQOL was in general very good in our study and that the IMPACT III (CH) may differently weigh variables of health. These findings may also indicate that patients and parents are prone to overestimate the value of CAM for their disease. Additionally, patients and parents' views of response to CAM correlated only to some extent. As in pediatrics, the treatment decisions mainly lie with the parents, parents may be biased in favor of these decisions. Therefore, it is important to perform high‐quality prospective studies with CAM to increase the knowledge of effective CAM as well as to guide physicians, patients and parents for the use of evidence‐based CAM therapies.

One limitation of our study is the limited number of patients, which did not allow us to investigate each CAM for CD and UC, as well as other subgroups, separately. With our cross‐sectional design we were able to give a snapshot of CAM use and the SIBDCS yearly follow‐ups allowed us to integrate some longitudinal data. However, longitudinal and interventional study designs would be needed to assess the benefit of different CAM on the disease course and to assess for example, for possible deficiencies due to dietary restrictions.

The availability of cohort data including HRQOL questionnaires allowed us to compare the view of the patients and parents with clinical data. As patients in the SIBDCS answer the IMPACT III (CH) yearly, bias is reduced for CAM users answering the IMPACT III (CH) more positively than no‐CAM users.

Additionally, with the inclusion of the CAM‐SD questionnaire into the yearly follow‐ups of the SIBDCS, we aimed to ensure comprehensive data collection and to minimize the potential for selection bias. As a result, our response rate of 60% exceeds the 10%–56% response rate of previously published studies.^44^, ^45^, ^46^

## 5 CONCLUSION

Our study shows that there is a great interest in CAM use in pediatric and adolescent IBD patients and their parents with a prevalence of 67%. Patients that adopted restricted diets are prone to use CAM. Physicians should be aware of these factors and include the risk of potential interactions and adverse events as well as resulting nutritional deficiencies in their treatment considerations and planning.

## CONFLICT OF INTEREST STATEMENT

The authors declare no conflicts of interest.

## Supporting information

Supporting information.

## Data Availability

The data and the questionnaire are available from the authors upon reasonable request.
