# Supplementary material for: Patterns of complementary and alternative medicine use in pediatric patients with inflammatory bowel disease
Source: J Pediatr Gastroenterol Nutr. 2025 Nov 6;82(1):84–92. doi: 10.1002/jpn3.70252 (PMC12780483; doi:10.1002/jpn3.70252)
Supplement: Supplementary file 1 — Supporting information. [file JPN3-82-84-s001.docx]

**Suppl. Table S1: Baseline characteristics of eligible patients in the Swiss IBD cohort study.**

| **Baseline characteristics** | **CAM-SD not available (n=72)** | **CAM-SD available (n=111)** | **p-value** |
| --- | --- | --- | --- |
| Age at diagnosis, years, median (IQR) | 11.0 (8.0, 13.0) | 10.0 (7.0, 13.0) | 0.240 |
| Sex: Female | 33 (46) | 54 (49) | 0.825 |
| Diagnosis |  |  | 0.550 |
| CD | 33 (36) | 59 (53) |  |
| UC | 29 (40) | 41 (37) |  |
| IBD-U | 10 (14) | 11 (10) |  |
| Linguistic region |  |  | 0.270 |
| French-speaking | 21 (30) | 43 (39) |  |
| German-speaking | 50 (70) | 68 (61) |  |

Comparison of baseline characteristics of patients where CAM-SD questionnaire was available and not available.

**Suppl. Table S2: Baseline characteristics of Crohn’s disease patients grouped by CAM use versus no-CAM use.**

| **Characteristics of CD patients** | **CAM (n=37)** | **No-CAM (n=22)** | **p-value** |
| --- | --- | --- | --- |
| Baseline | | | |
| Age at diagnosis, years, median (IQR) | 11.0 (8.0, 13.0) | 11.5 (10.0, 14) | 0.321 |
| Gender: Female | 17 (46) | 13 (59) | 0.329 |
| Disease location at diagnosis  L1  L2  L3  L4  Missing | 2 (5.6)  8 (22)  21 (58)  5 (14)  1 (3) | 1 (5.0)  3 (15)  12 (60)  4 (20)  2 (9) | 0.873 |
| Penetrating disease | 8 (22) | 10 (45) | 0.3 |
| Stenosing disease | 4 (11) | 2 (9) | 1.0 |
| IBD related surgery | 4 (11) | 3 (14) | 1.0 |
| Extraintestinal manifestations | 24 (65) | 11 (50) | 0.261 |
| Family history of IBD | 4 (11) | 3 (14) | >0.999 |
| **At CAM-SD questionnaire:** | | | |
| Age, years, mean (IQR) | 14.0 (13.0, 16.0) | 14.5 (14.0, 16.0) | 0.455 |
| Disease duration, years, median (IQR) | 3.0 (2.0, 4.0) | 3.0 (2.2, 4.0) | 0.424 |
| Disease activity (wPCDAI)  Remission  Mild disease  Moderate disease  Severe disease  Missing | 21 (75)  5 (18)  1 (3.6)  1 (3.6)  9 (24) | 15 (94)  1 (6.2)  0 (0)  0 (0)  6 (27) | 0.586 |
| Dietary restrictions | 24 (65) | 5 (23) | 0.002 |
| HRQOL |  |  |  |
| IMPACT III (CH) total score, median (IQR) | 98 (91, 104) | 98 (92, 99) | 0.629 |

The weighted pediatric Crohn’s disease activity index (wPCDAI) was used to assess disease activity in pediatric CD patients with the following categories: remission (<12.5), mild disease (≥12.5 - 42), moderate disease (≥42.5 - 57), severe disease (≥57.5).

**Suppl**. **Table S3: Baseline characteristics of UC/IBD-U patients grouped by CAM use versus no-CAM use.**

| **Characteristics of UC/IBD-U patients** | **CAM (n=35)** | **No-CAM (n=17)** | **p-value** |  |
| --- | --- | --- | --- | --- |
| Baseline | | | |  |
| Diagnosis  Ulcerative colitis  IBD-U | 25 (71)  10 (29) | 16 (94)  1 (6) | 0.078 |  |
| Age at diagnosis, years, median (IQR) | 9.0 (4.0, 12.0) | 8.0 (6.0, 13.0) | >0.999 |  |
| Gender: Female | 16 (46) | 8 (47) | 0.927 |  |
| Disease location at diagnosis  E2  E4  Missing | 7 (21)  26 (79)  2 (6) | 5 (31)  11 (69)  1 (6) | 0.492 |  |
| Surgery (Colectomy) | 1 (3) | 1 (6) | 0.5 |  |
| Extraintestinal manifestations | 13 (37) | 7 (41) | 0.779 |  |
| Family history of IBD | 2 (6) | 4 (24) | 0.081 |  |
| **At CAM-SD questionnaire:** | | | | |
| Age, years, mean (IQR) | 15.00 (12.00, 16.00) | 13.00 (12.00, 16.00) | 0.634 |  |
| Disease duration, years, median (IQR) | 4 (3, 7) | 4 (3, 5) | 0.459 |  |
| Disease activity (PUCAI)  Remission  Mild disease  Moderate disease  Missing | 28 (82)  4 (12)  2 (6)  1 (3) | 12 (86)  1 (6)  1 (6)  3 (18) | >0.999 |  |
| Dietary restrictions | 24 (69) | 6 (35) | 0.023 |  |
| HRQOL  IMPACT III (CH) total score, median (IQR) | 98 (90, 103) | 97 (90, 102) | 0.976 |  |

Pediatric ulcerative colitis activity index (PUCAI) was used to assess disease activity in pediatric UC/IBD-U patients with the following categories: remission (≤10), mild disease (11-34), moderate disease (35-64), severe disease (≥65).

**Suppl. Table S4: Use of complementary medicine.**

|  | **Currently**  **N** | **Previously**  **N** |
| --- | --- | --- |
| *In context of your IBD, did you use …* | | |
| **Manual therapies:** |  |  |
| Osteopathy | 0 | 14 |
| Massage | 6 | 11 |
| Reflexology | 1 | 4 |
| Chiropractor | 1 | 1 |
| Shiatsu | 0 | 1 |
| **Energy-based therapies:** |  |  |
| Homeopathy | 1 | 21 |
| Bach flowers | 1 | 7 |
| Spagyrika | 2 | 6 |
| Bioresonance | 0 | 7 |
| **Traditional medicine:** |  |  |
| TCM | 1 | 6 |
| Ayurvedic medicine | 0 | 1 |
| **Other categories:** |  |  |
| Naturopathy | 5 | 15 |
| Kinesiology | 3 | 8 |
| Acupuncture | 0 | 8 |
| **Mental therapies**: |  |  |
| Psychotherapy | 8 | 13 |
| Hypnosis | 1 | 7 |
| Meditation | 0 | 8 |
| Cognitive behaviour therapy | 1 | 0 |
| Autogenic training | 1 | 0 |
| Mindfulness | 0 | 1 |
| Yoga | 0 | 1 |
| other | 3 | 2 |
| **Herbal medicine**: |  |  |
| Curcumin | 6 | 9 |
| Boswellia serra extract | 0 | 4 |
| Aloe vera | 0 | 2 |
| Plantago ovata | 0 | 2 |
| Cannabis (THC) for medical reasons | 1 | 1 |
| Pure peppermintoil | 0 | 1 |
| Peppermintoil mixture | 0 | 1 |
| Artemisia absinthum | 0 | 1 |
| other | 5 | 10 |

Use of manual therapies, energy-based therapy, traditional Chinese medicine, mental therapies and herbal medicine in Swiss pediatric IBD patients. Number of patients who used a specific treatment/supplement are provided. More than one answer per patient was possible and patients might have used the specific CAM currently and/or previously.

**Suppl. Table S5**: **Overview of micronutrient, vitamin, supplement and probiotic use.**

|  | **Currently**  **N (%)** | **Previously**  **N (%)** | **Self-medication**  **N (%)** | **With prescription**  **N (%)** | **Not specified whether self-medication or with prescription**  **N (%)** |
| --- | --- | --- | --- | --- | --- |
| *In context of your IBD, did you take …* |  |  |  |  |  |
| **Vitamins and supplements** |  |  |  |  |  |
| Multivitamins | 11 | 17 | 16 | 5 | 3 |
| Vitamin B12 | 4 | 5 | 3 | 4 | 1 |
| Vitamin C | 5 | 5 | 4 | 4 | 2 |
| Vitamin D | 40 | 46 | 9 | 60 | 2 |
| Vitamin E | 1 | 1 | 1 | 1 | 0 |
| Folic acid | 9 | 4 | 2 | 9 | 0 |
| Omega 3, fishoil | 6 | 11 | 9 | 6 | 0 |
| Others | 4 | 4 | 3 | 3 | 0 |
| **Micronutrients** |  |  |  |  |  |
| Iron (oral) | 6 | 45 | 2 | 43 | 3 |
| Calcium | 7 | 21 | 2 | 21 | 0 |
| Potassium | 0 | 2 | 1 | 0 | 0 |
| Magnesium | 2 | 14 | 6 | 7 | 0 |
| Other | 3 | 4 | 2 | 4 | 0 |
| **Products to help digestion** |  |  |  |  |  |
| Lactase | 0 | 3 | 2 | 1 | 0 |
| Pancreatic enzymes | 0 | 1 | 0 | 1 | 0 |
| Other | 6 | 2 | 2 | 6 | 3 |
| **Probiotics** |  |  |  |  |  |
| Enterococcus SF 68 | 0 | 16 | 6 | 10 | 0 |
| Saccharomyces boulardii | 4 | 8 | 2 | 9 | 0 |
| E.coli Nissle | 3 | 7 | 0 | 9 | 0 |
| Other | 4 | 9 | 7 | 5 | 0 |

Numbers of patients who took a specific vitamin/supplement are provided. Patients might have taken the product currently and/or previously.

**Suppl. Table S6: Reasons of CAM use, cost coverage, source of information and communication.**

|  | N (%) |
| --- | --- |
| *What were your reasons for CAM? (multiple answers possible)* |  |
| Enforcement of immune system | 20 (28) |
| Could help, no side effects | 40 (56) |
| Recommended by physician | 18 (25) |
| Recommended by friends/family | 12 (17) |
| Cost-efficient | 1 (1) |
| Easy availability | 0 (0) |
| Other reasons | 13 (18) |
| *Did you pay for the complementary treatment yourself?* |  |
| Yes, completely | 29 (32) |
| Yes, partially | 30 (33) |
| No, insurance covered the costs | 31 (34) |
| *Did you inform your treating physician about your use of CAM?* |  |
| Yes | 37 (51) |
| Yes, partially | 18 (25) |
| No | 9 (12) |
| Do not know | 2 (3) |
| *How and where do you obtain information on complementary medicine treatment options?* |  |
| Family | 27 (37) |
| Friends/neighbours | 19 (26) |
| Internet/social media | 27 (37) |
| General practitioner | 14 (19) |
| Support group | 6 (8) |
